# Drug-related problems associated with direct oral anticoagulants: an observational cross-sectional study of medical record review by pharmacists in a large teaching hospital

**DOI:** 10.1016/j.rpth.2024.102354

**Published:** 2024-02-15

**Authors:** Melissa M.Y. Chow, Duke J.J. Chua, Vivian S.Y. Wong, Sin Y. How, Sei K. Koh, Xin Y. Tay, Lai H. Lee

**Affiliations:** 1 Pharmacy Department, Singapore General Hospital, Singapore; 2 Haematology Department, Singapore General Hospital, Singapore

**Keywords:** anticoagulants, direct oral anticoagulants, drug-related problems, interventions, pharmacist review

## Abstract

**Background:**

Prescribing DOACs presents with challenges in the elderly and patients with renal and hepatic impairment. To mitigate safety risks, pharmacists have a role in detection, prevention, and resolution of DOAC-associated drug-related problems (DRPs).

**Objectives:**

To identify the types of DOAC-associated DRPs in patients on DOAC therapy and factors that predispose patients to DOAC-associated DRPs.

**Methods:**

An observational cross-sectional study was conducted in SGH from January 1, 2017, to May 31, 2019, on patients prescribed with a DOAC (rivaroxaban, dabigatran, and apixaban). Data were electronically extracted for patient demographics, clinical characteristics, and details of DOAC-related DRPs identified by pharmacists. Matching of DRP group to non-DRP group at a ratio of 1:2 based on gender, race, and DOAC was performed. The DRP group included patients with detected DRPs while non-DRP group included patients without them. Descriptive analysis was used to summarize patient characteristics and types of DOAC-associated DRPs. In the matched population, conditional logistic regression was used to calculate unadjusted (UOR) and adjusted odds (AOR) ratio to detect association of DOAC-associated DRPs with age, renal function, ≥2 comorbidities, and DOAC indication (atrial fibrillation [AF] vs venous thromboembolism).

**Results:**

A total of 8432 patients prescribed DOACs were analyzed, which consisted of 827 (9.8%) and 7602 (90.2%) patients with DRPs and no DRPs, respectively. The top DOAC-associated DRP was inappropriate drug regimen (*n* = 487, 60.1%). After matching, 2403 patients were analyzed, consisting of 801 patients from DRP group and 1602 from non-DRP group. Factors associated with DOAC-associated DRPs were statistically significant for renal function at creatinine clearance (CrCl) of >30 to 50 mL/min/1.73 m^2^ (AOR: 1.42; 95% CI: 1.14-1.76; *P* = .002), 15 to 30 mL/min/1.73 m^2^ (OR: 1.94; 95% CI: 1.42-2.66; *P* < .001), and <15 mL/min/1.73m^2^ (OR: 2.35; 95% CI: 1.13-4.88; *P* = .022), respectively, compared with a CrCl of >50 mL/min/1.73 m^2^ and DOAC indication for AF (AOR: 1.84; 95% CI: 1.47-2.30; *P* < .001) compared with venous thromboembolism.

**Conclusion:**

Inappropriate drug regimen was the most common DOAC-associated DRP. Impaired renal function and patients with AF increased the likelihood of DOAC-associated DRPs.

## Essentials

• Pharmacists are crucial in detecting and resolving errors from prescribing direct oral anticoagulants (DOACs).
• All DOAC prescriptions were reviewed by pharmacists in a large hospital and prescribing errors were recorded.
• The common prescribing errors were dose-related, avoidance of adverse events, and drug interactions.
• Patients with renal dysfunction and on DOAC for atrial fibrillation were associated with prescription errors.

## 1 Introduction

Direct oral anticoagulants (DOACs) were favored by clinicians as the armamentarium for managing thrombotic conditions such as venous thromboembolism (VTE), atrial fibrillation (AF), venous thromboprophylaxis, and cancer-associated thrombosis. Compared with warfarin, DOACs require less frequent monitoring and have fewer drug–drug and drug–food interactions, fixed dose regimens, and favorable safety profile with lower rates of intracranial hemorrhage [1]. Anticoagulants have been associated with preventable adverse drug events (ADEs) such as bleeding and thrombotic complications [2]. The risk of ADEs from DOACs were higher in patients with renal or hepatic impairment, the elderly, or those taking concurrent interacting drugs [3]. Pharmacists can play a collaborative role in preventing ADEs with DOACs by identifying and resolving DOAC-associated drug-related problems (DRPs) during the medication delivery process [4].

The Pharmaceutical Care Network Europe (PCNE) defined DRP as an event or circumstance involving drug therapy that actually or potentially interferes with desired health outcomes [5]. Prescribing DOACs presents with unique clinical challenges, which can lead to inadvertent prescribing errors and poor outcomes. DOACs require dose adjustment in patients with renal impairment and should be avoided in liver cirrhosis Child–Pugh classification B or C. Dose adjustments should be considered in the elderly (aged >80 years). There is safety and efficacy uncertainty for DOACs prescribed to patients with extremes in body weight (<60 kg or >120 kg). Concomitant use of DOACs with coexisting medications that are potent inhibitors or inducers of hepatic cytochrome P450 enzymes or p-glycoprotein transporters should be avoided as this can lead to supratherapeutic or subtherapeutic levels of DOACs in the body, potentially increasing risk of bleeding or thrombosis, respectively [6].

Many studies have highlighted the challenges in prescribing DOACs, and DOAC-associated DRPs resulting from prescribing errors were commonly detected [7,8]. DRPs are undesirable and can lead to increased morbidity, mortality, and healthcare costs [9]. Implications of ADEs from DOAC-associated DRPs can be catastrophic leading to bleeding or thrombotic events [10]. To mitigate safety risks of DOAC prescription, pharmacists have an essential role in detection, prevention, and resolution of DOAC-associated DRPs [11].

As the anticoagulation paradigm shifts toward DOACs with its desirable characteristics and increasing number of approved indications, DOAC prescriptions will inevitably increase. Insight into the causes of prescribing errors will enable development of strategies to reduce prescribing errors and DRPs and prevent ADEs. There is a dearth of studies looking at prescribing patterns of DOACs in the Asian population. This study aims to look at the types of DOAC-associated DRPs from prescribing rivaroxaban, dabigatran, and apixaban and identify factors linked to DRPs in a large academic hospital in Asia.

## 2 Methods

### 2.1 Study design and ethics review

This was a single-center cross-sectional study conducted in Singapore General Hospital in patients prescribed with DOACs from January 1, 2017, to May 31, 2019. The study was part of the quality improvement project in DOAC-prescribing habits. Nevertheless, the study was submitted to institution’s ethics review board and exempted from review.

### 2.2 Study objectives

To examine the distribution and types of DOAC-associated DRPs among adults prescribed DOAC therapy, and identify factors that predispose patients to DOAC-associated DRPs.

### 2.3 Study population and data acquisition

Data of adults aged 21 years with DOAC prescription were extracted from electronic medical records. Data included patient demographics (race, gender, and age) and clinical characteristics (body weight, chronic conditions, renal function, and DOAC prescriptions such as the type of DOAC and dose). The DOAC prescription and clinical characteristics prior to time of intervention were captured.

Factors that predisposed patients to DRPs were age, renal function, the presence of comorbidities (dichotomized into <2 and ≥2 comorbidities), and DOAC indication (AF vs VTE). These factors were recognized by previous studies to be associated with DRPs [12]. Renal function was calculated using the Cockcroft–Gault equation based on actual body weight.

### 2.4 Pharmacist interventions and DRP classification

A pharmacist intervention is defined as “a recommendation initiated by the pharmacist in response to a DRP during any phase of the medication supply process” [5]. All drug orders required mandatory review by pharmacists prior to supply or patient administration as part of the institution’s safety policy. The DOAC orders were reviewed for compliance to licensed recommendations from Health Science Authority of Singapore (Supplementary Table S1). The pharmacists were alerted to deviations from labeled dose or indication as well as patients’ clinical condition (eg. bleeding) that contradicted use of DOAC that could potentially lead to negative outcomes. The DRPs were addressed by the pharmacist by providing recommendations to the prescriber to change the drug order.

Data of all DOAC-associated interventions were electronically extracted (DOAC, types of DRPs, recommendations, intervention outcomes, and prescriber disciplines). The DRPs were classified according to modified PCNE DRP classification. Any uncertainty in the classification of DRPs were discussed among the coinvestigators and consensus was reached based on the modified PCNE DRP definitions [5]. The definitions for DRPs classifications and examples are displayed in Supplementary Table S2.

### 2.5 DRP group and non-DRP group matching

The DRP group and non-DRP groups were compared with look for factors predisposing patients to DOAC-associated DRPs. Matching DRP group to non-DRP group at a ratio of 1:2 was performed based on gender, race, and type of DOAC. This ensured even distribution of confounding factors such as gender, race, and the DOAC prescribed among the 2 groups. The DRP group included patients with detected DRPs who received interventions by pharmacists while non-DRP group included patients with no DRPs detected and no interventions performed. Patients from the non-DRP group were considered once for each matched patient from the DRP group. A 1:2 ratio was chosen because the power of the study to detect a difference in the proportion of patients in DRP group between any pair of DOACs seemed to taper off at this ratio.

### 2.6 Sample size calculation

Sample size was calculated based on 95% CI, alpha error of 5%, and power of 80% to detect odds ratio of at least 1.5 for factors linked with DOAC-associated DRPs. Based on DRP group to non-DRP group matching ratio of 1:2, the minimum total sample size was calculated to be 876, with 292 for DRP group and 584 for non-DRP group [13].

### 2.7 Statistical analysis

Descriptive analyses were used to summarize patient demographics, types, and distribution of DOAC-associated DRPs. Numbers (percentages) were presented for categorical data and mean values (SD) were presented for continuous data depending on the data distribution. To identify factors associated with DOAC-associated DRPs in the unmatched study population, a binomial logistic regression was used to calculate the unadjusted and adjusted odds ratio (adjusted for age, renal function, number of comorbidities, and DOAC indication). Similarly, a conditional logistic regression was used in the matched study population. Statistical significance was achieved when the 2-sided *P* value was < .05. Statistical analysis was performed using Statistical Package for Social Sciences (SPSS, 28th edition).

### 2.8 Handling of missing data

A total of 326 (3.9%) patients had missing data for this study, which was managed using the mean substitution method where in place of the missing data, the mean value of the same variable was used [14]. Missing data included the weight (*n* = 254) or the serum creatinine (*n* = 72). Mean substitution was used to prevent patients with missing data being omitted from analysis, which may affect the study power.

## 3 Results

From January 1, 2017, to May 31, 2019, a total of 8432 patients prescribed with DOACs were extracted, of which 827 (9.8%) patients had DOAC-associated DRPs and interventions performed. Among the patients prescribed DOACs, the most common ones to receive an intervention were patients on rivaroxaban (*n* = 613/4130, 14.8%) followed by dabigatran (*n* = 42/384, 10.9%) and apixaban (*n* = 172/3918, 4.4%). The average age for patients on DOAC therapy was 70 (±13) years and the top 2 indications for DOACs in both the DRP and non-DRP groups were AF (*n* = 590, 71.3%; *n* = 5045, 66.3%), followed by VTE (*n* = 180, 21.8%; n = 2221, 29.1%), respectively (Table 1).

**Table 1:** Patient demographics and indications of unmatched population.

Patient demographics	Total *n* (%) 8432		Rivaroxaban 4130 (49)		Dabigatran 384 (4.6)		Apixaban 3918 (46.4)	
	DRP group 827 (9.8)	Non-DRP group 7605 (90.2)	DRP group 613 (14.8)	Non-DRP group 3517 (85.2)	DRP group 42 (10.9)	Non-DRP group 342 (89.1)	DRP group 172 (4.4)	Non-DRP group 3746 (95.6)
Gender								
Male	446 (53.9)	4256 (56)	326 (53.2)	1932 (54.9)	21 (50)	201 (58.8)	99 (57.6)	2123 (56.7)
Female	381 (46.1)	3349 (44)	287 (46.8)	1585 (45.1)	21 (50)	141 (41.2)	73 (42.4)	1623 (43.3)
Race								
Chinese	617 (74.6)	5905 (77.6)	464 (75.7)	2662 (75.7)	27 (64.3)	266 (77.8)	126 (73.3)	2977 (79.5)
Malay	116 (14)	683 (9)	80 (13.1)	339 (9.6)	7 (16.7)	20 (5.8)	29 (16.9)	324 (8.6)
Indian	63 (7.6)	464 (6.1)	49 (8)	219 (6.2)	3 (7.1)	15 (4.4)	11 (6.4)	230 (6.1)
Others	31 (3.7)	553 (7.3)	20 (3.3)	297 (8.4)	5 (11.9)	41 (12)	6 (3.5)	215 (5.7)
Mean age (years ± SD)	70 ± 13	69 ± 13	70 ± 13	67 ± 13	68 ± 13	68 ± 13	72 ± 12	72 ± 12
Age (years)								
21-50	56 (6.8)	569 (7.5)	43 (7)	376 (10.7)	6 (14.3)	26 (7.6)	7 (4.1)	167 (4.5)
51-65	179 (21.6)	1813 (23.8)	144 (23.5)	950 (27.0)	8 (19)	82 (24)	27 (15.7)	781 (20.8)
66-75	222 (26.8)	2345 (30.8)	153 (25)	1134 (32.2)	12 (28.6)	114 (33.3)	57 (33.1)	1097 (29.3)
>75	370 (44.7)	2878 (37.8)	273 (44.5)	1057 (30.1)	16 (38.1)	120 (35.1)	81 (47.1)	1701 (45.4)
Comorbidities								
≥2	481 (58.2)	4100 (53.9)	354 (57.7)	1574 (44.8)	22 (52.4)	140 (40.9)	105 (61)	2386 (63.7)
<2	346 (41.2)	3505 (46.1)	259 (42.3)	1943 (55.2)	20 (47.6)	202 (59.1)	67 (39)	1360 (36.3)
Indication								
AF	590 (71.3)	5045 (66.3)	405 (66.1)	1903 (54.1)	26 (61.9)	225 (65.8)	159 (92.4)	2917 (77.9)
VTE	180 (21.8)	2221 (29.2)	160 (26.1)	1444 (41.1)	12 (28.6)	107 (31.3)	8 (4.7)	670 (17.9)
Valvular AF	12 (1.5)	27 (0.4)	10 (1.6)	12 (0.3)	1 (2.4)	1 (0.3)	1 (0.6)	14 (0.4)
PVD	14 (1.7)	241 (3.2)	14 (2.3)	112 (3.2)	0 (0)	5 (1.5)	0 (0)	124 (3.3)
ACS-related	5 (0.6)	45 (0.6)	5 (0.8)	30 (0.9)	0 (0)	0 (0)	0 (0)	15 (0.4)
Others	26 (3.1)	26 (0.3)	19 (3.1)	16 (0.5)	3 (7.1)	4 (1.2)	4 (2.3)	6 (0.2)

ACS, acute coronary syndrome; AF, atrial fibrillation; DRP, drug-related problem; PVD, peripheral vascular disease; VTE, venous thromboembolism.

**Figure 1:**
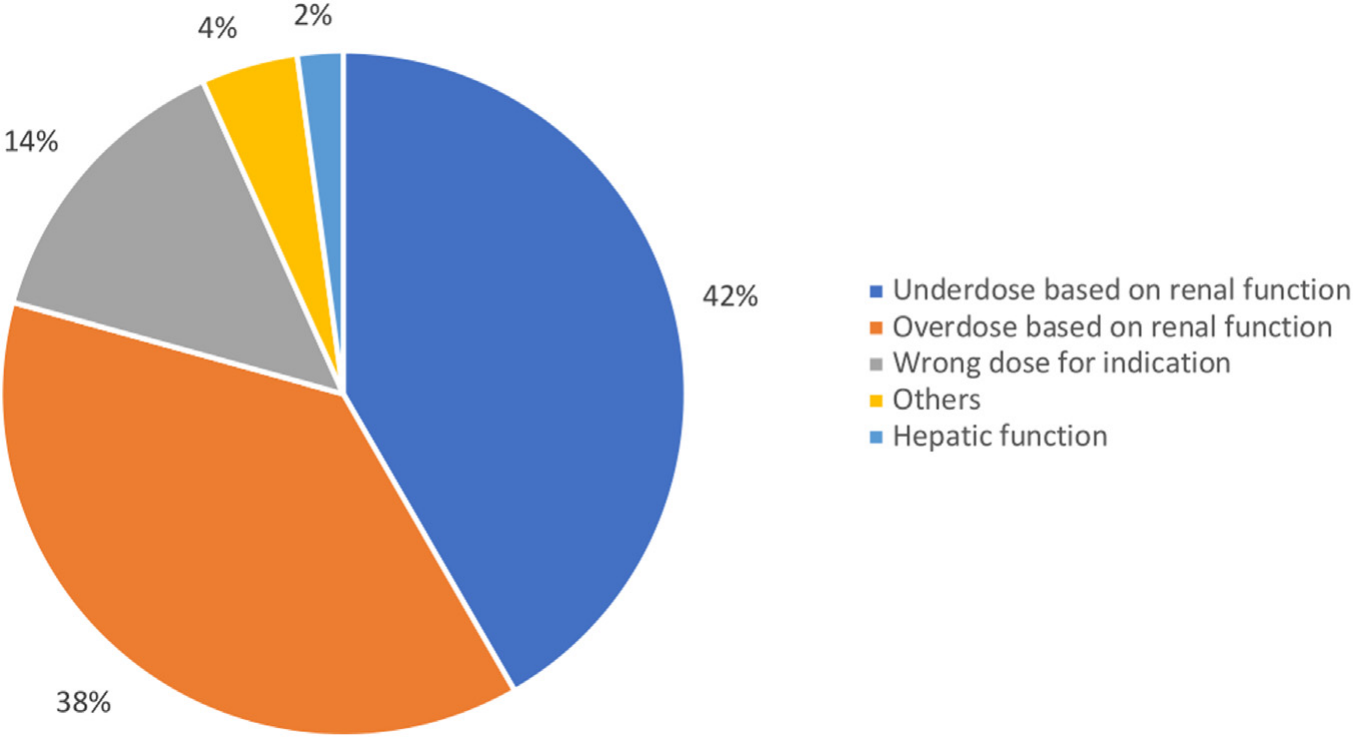
Distribution of types of inappropriate drug regimen based on dose.

Among the patients with DRPs, the most common DRPs were inappropriate drug regimen (*n* = 497, 60.1%) followed by avoidance of adverse events (*n* = 102, 12.3%) and drug interactions (*n* = 52, 6.3%). A subanalysis of the inappropriate drug regimen showed that inappropriate drug regimen based on dose was the most common DRP among all 3 DOACs (*n* = 372, 74.8%) (Table 2). Figure 1 showed that the most prevalent reasons for inappropriate dose were underdosing by renal function (*n* = 155, 41.7%) followed by overdosing by renal function (*n* = 140, 37.6%) and wrong dose for indication (*n* = 52, 14%).

**Table 2:** Distribution of DRPs by type and DOAC in the DRP group.

Type of DRPs	Total *n* (%) 827	Rivaroxaban 613 (74.1)	Dabigatran 42 (5.1)	Apixaban 172 (20.2)
Inappropriate drug regimen^a^	497 (60.1)	391 (63.8)	18 (42.9)	88 (52.2)
Dose	372 (74.8)	289 (73.9)	10 (55.6)	73 (83)
Frequency	20 (4)	15 (3.8)	1 (5.6)	4 (4.5)
Duration	62 (12.5)	56 (14.3)	4 (22.2)	2 (2.3)
Route	2 (0.4)	1 (0.3)	1 (5.6)	0 (0)
Instruction	18 (3.6)	14 (3.6)	1 (5.6)	3 (3.4)
Omission of drug	31 (3.7)	17 (4.3)	4 (22.2)	10 (11.4)
Improper drug selection	42 (5.1)	27 (4.4)	8 (19)	7 (4.1)
Therapeutic duplication	37 (4.5)	28 (4.6)	3 (7.1)	6 (3.5)
No indication	11 (1.3)	8 (1.3)	0 (0)	3 (1.7)
Avoidance of adverse event	102 (12.3)	72 (11.7)	6 (14.3)	24 (14)
Therapeutic substitution	4 (0.5)	4 (0.7)	0 (0)	0 (0)
Drug interaction	52 (6.3)	35 (5.7)	1 (2.4)	16 (9.3)
Monitoring parameter recommendation	8 (1)	4 (0.7)	1 (2.4)	3 (1.7)
Clarification of drug order	25 (3)	17 (2.8)	1 (2.4)	7 (4.1)
Others	18 (2.2)	10 (1.6)	0 (0)	8 (4.7)

DRP, drug-related problem.

a Twenty-three missing inappropriate drug regimen categories.

**Figure 2:**
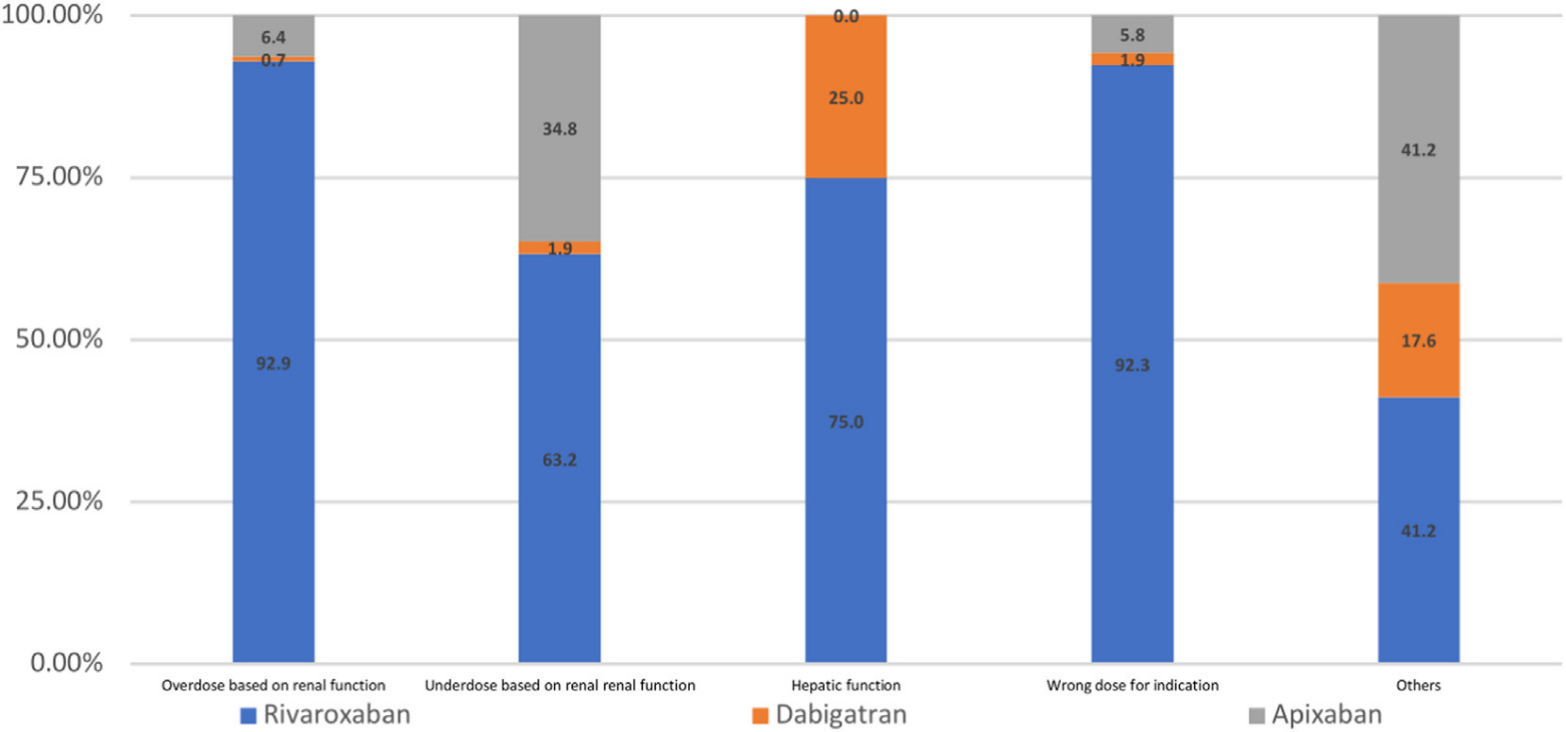
Distribution of inappropriate drug regimen based on dose among the DOACs.

**Figure 3:**
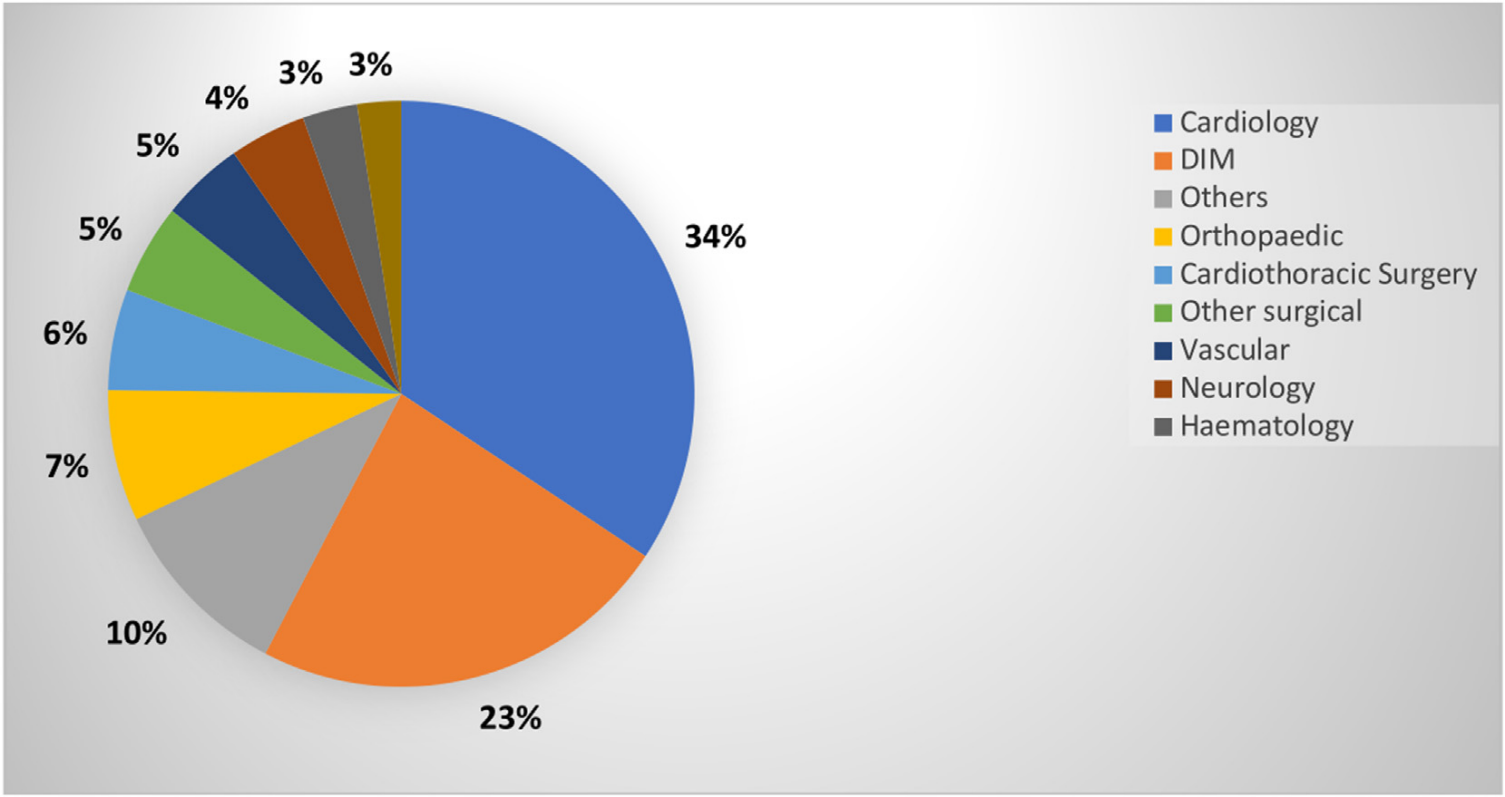
Distribution of interventions based on discipline.

Figure 2 showed that rivaroxaban (92.9%) was the most implicated DOAC associated with overdosing by renal function followed by apixaban (6.4%). Rivaroxaban (63.2%) was the most common DOAC implicated for underdosing by renal function, followed by apixaban (34.8%). Apixaban was more commonly implicated for DRP due to underdosing by renal function compared with overdose.

After matching for confounders (gender, race, and DOAC), a total of 2403 patients on DOACs were analyzed. The DRP group consisted of 801 patients and the non-DRP group consisted of 1602 patients. The number of patients on rivaroxaban, dabigatran, and apixaban in the control group was reduced by 66.6% (from 3517 to 1176), 76% (from 342 to 82), and 90.8% (from 3746 to 344), respectively, after matching (Table 3). The average age for patients on all 3 DOACs was similar to that of the unmatched population (70 ± 13 years), and the majority were on DOACs for AF (64.3%) and VTE (43.2%) (Table 3).

**Table 3:** Patient demographics for matched population (matched 1:2).

Patient demographics	Total *n* (%) 2403		Rivaroxaban 1764 (73.4)		Dabigatran 123 (5.1)		Apixaban 516 (21.5)		*P* value
	DRP group 801	Non-DRP group 1602	DRP group 588	Non-DRP group 1176	DRP group 41	Non-DRP group 82	DRP group 172	Non-DRP group 344	NS
Gender									NS
Male	436 (54.4)	872 (54.4)	316 (53.7)	632 (53.7)	21 (51.2)	42 (51.2)	99 (57.6)	198 (57.6)	
Female	365 (45.6)	730 (45.6)	272 (46.3)	544 (46.3)	20 (48.8)	40 (48.8)	73 (42.4)	146 (42.4)	
Race									NS
Chinese	603 (75.3)	1206 (75.3)	450 (76.5)	900 (76.5)	27 (65.9)	54 (65.9)	126 (73.3)	252 (73.3)	
Malay	108 (13.5)	216 (13.5)	73 (12.4)	146 (12.4)	6 (14.6)	12 (14.6)	29 (16.9)	58 (16.9)	
Indian	59 (7.4)	118 (7.4)	45 (7.7)	90 (7.7)	3 (7.3)	6 (7.3)	11 (6.4)	22 (6.4)	
Others	31 (3.9)	62 (3.9)	20 (3.4)	40 (3.4)	5 (12.2)	10 (12.2)	6 (3.5)	12 (3.5)	
Mean age ± SD	70 ± 13	69 ± 12	70 ± 13	68 ± 13	68 ± 13	69 ± 12	72 ± 12	72 ± 11	
Age (years)									
21-50	53 (6.6)	115 (7.2)	40 (6.8)	97 (8.2)	6 (14.6)	6 (7.3)	7 (4.1)	12 (3.5)	
51-65	174 (21.7)	387 (24.2)	139 (23.6)	295 (25.1)	8 (19.5)	18 (22)	27 (15.7)	74 (21.5)	
66-75	218 (27.2)	548 (34.2)	150 (25.5)	411 (34.9)	11 (26.8)	26 (31.7)	57 (33.1)	111 (32.3)	
>75	356 (44.4)	552 (34.5)	259 (44)	373 (31.7)	16 (39)	32 (39)	81 (47.1)	147 (42.7)	
Comorbidities									
≥2	461 (57.6)	809 (50.5)	334 (56.8)	549 (46.7)	22 (53.7)	37 (45.1)	105 (61)	223 (64.8)	
<2	340 (42.4)	793 (49.5)	254 (43.2)	627 (52.3)	19 (46.3)	45 (54.9)	67 (39)	121 (35.2)	
Indication									
AF	572 (71.4)	973 (60.7)	387 (65.8)	651 (55.4)	26 (63.4)	62 (75.6)	159 (92.4)	260 (75.6)	
VTE	174 (21.7)	565 (35.3)	155 (26.4)	475 (40.4)	11 (26.8)	19 (23.2)	8 (4.7)	71 (20.6)	
Valvular AF	12 (1.5)	2 (0.1)	10 (1.7)	2 (0.2)	1 (2.4)	0 (0)	1 (0.6)	0 (0)	
PVD	12 (1.5)	51 (3.2)	12 (2)	38 (3.2)	0 (0)	0 (0)	0 (0)	13 (3.8)	
ACS-related	5 (0.6)	6 (0.4)	5 (0.9)	6 (0.5)	0 (0)	0 (0)	0 (0)	0 (0)	
Others	26 (3.2)	5 (0.3)	19 (3.2)	4 (0.3)	3 (7.3)	1 (1.2)	4 (2.3)	0 (0)	

ACS, acute coronary syndrome; AF, atrial fibrillation; DRP, drug-related problem; NS, not significant; PVD, peripheral vascular disease; VTE, venous thromboembolism.

Figure 3 showed that majority of interventions for DOAC-associated DRPs originated from drug orders by cardiology (*n* = 284, 34%) and internal medicine (*n* = 193, 23%) disciplines.

Table 4 showed the unadjusted and adjusted odds ratio for the factors associated with DRPs for the matched populations. The adjusted odd ratios for both age and the presence of 2 or more comorbidities were not statistically significantly. The adjusted odd ratios for renal function was statistically significant for creatinine clearance of >30 to 50 mL/min/1.73 m^2^ (OR: 1.42; 95% CI: 1.14-1.76; *P* = .002), 15 to 30 mL/min/1.73 m^2^ (OR: 1.94; 95% CI: 1.42-2.66; *P* < .001), and <15 mL/min/1.73 m^2^ (OR: 2.35; 95% CI: 1.13-4.88; *P* *=* .022) between the DRP and non-DRP groups, respectively. When comparing patients with VTE and AF between the DRP and non-DRP groups, the adjusted odd ratios was statistically significant (OR: 1.84; 95% CI: 1.47-2.30; *P* < .001). A sensitivity analysis was done for the unmatched populations, which showed similar trend (Supplementary Table S6).

**Table 4:** Conditional logistic regression for matched population (1:2) for factors associated with DRPs.

*n* = 2403 DRP group = 801 Non-DRP group = 1602	Unadjusted		Adjusted	
Factor	OR (95% CI)	*P* value	AOR (95% CI)	*P* value
Age (years)				
21-50	Reference		Reference	
51-65	1.00 (0.68-1.45)	.98	0.96 (0.66-1.40)	.82
66-75	0.88 (0.61-1.27)	.50	0.76 (0.53-1.11)	.16
>75	1.46 (1.01-2.09)	.04	1.09 (0.74-1.59)	.66
CrCl (mL/min/1.73 m^2^)				
>50	Reference		Reference	
>30 to 50	1.53 (1.25-1.87)	<.001	1.42 (1.14-1.76)	.002
15 to 30	2.24 (1.67-3.00)	<.001	1.94 (1.42-2.66)	<.001
<15	2.44 (1.18-5.02)	.02	2.35 (1.13-4.88)	.02
≥2 comorbidities				
Yes	1.33 (1.12-1.58)	.001	1.15 (0.96-1.39)	.12
Indication				
AF vs VTE	2.00 (1.62-2.48)	<.001	1.84 (1.47-2.30)	<.001

AF, atrial fibrillation; CrCl, creatinine clearance; DRP, drug-related problem; VTE, venous thromboembolism.

## 4 Discussion

This study gave insight into the types of DOAC-associated DRPs identified in an academic medical center and the factors that predisposed patients to such DRPs. Our study identified that inappropriate drug regimen was the most common DOAC-associated DRP, followed by avoidance of adverse events and drug interaction. Among the 3 DOACs, patients on rivaroxaban had the highest proportion of interventions, while patients on apixaban had the least. Subanalysis of the matched populations showed that patients with poor renal function and AF were more likely linked to DOAC-associated DRPs.

Our study detected 9.8% prevalence of DRPs among patients on DOACS and observed that DRPs were more prevalent in patients on rivaroxaban, followed by dabigatran and apixaban. This was comparable to a study by Viprey et al., which found prevalence of 8.4% of DRPs from DOACs among hospitalized patients and identified that patients on rivaroxaban for AF had the highest prevalence of DRPs when compared with other DOACs [15]. Furthermore, DOAC-associated DRPs were frequent in hospitalized patients, which was a similar finding to our study wherein 92% of DRPs were identified in the inpatient setting.

This study observed that the most common DOAC-associated DRP was related to dosing. This corroborated with other studies, which identified that inappropriate dosing of DOACs was the most prevalent DRP [15,16]. The sub analysis of inappropriate dose found that underdosing due to renal function was identified as the most common cause of inappropriate drug regimen, followed by overdose based on renal function and wrong dose for indication. Similarly, a meta-analysis study of DOAC-related prescribing errors revealed that common prescribing errors were dose-related, contributed by lack of adjustment for renal function, wrong indication, advanced age, and altered weight [17].

Among the recommendations made by the pharmacists, 68% were accepted by the physicians. This was within the acceptance rates of 60% to 80% in most studies where pharmacists were incorporated into the medical team to review medication orders [[18], [19], [20]]. There were some reasons postulated in which 27% of recommendations were not accepted or partially accepted. We elucidated that majority of DRPs addressing suboptimal doses of DOACs were not accepted because physicians deliberately intended to reduce doses due to concern with increased bleeding risks especially in the setting of reinitiating DOACs postoperatively when hemostasis was not established, or in elderly, frail, or underweight patients. Clear communication on physicians’ prescribing intent will prevent the pharmacist inadvertently detecting the order as a DRP and putting forth unnecessary recommendations.

Dose adjustments for DOACs were complicated and multifactorial. Renal dose adjustment for DOACs was also dependent on the indication. For patients with AF, the renal threshold to adjust for rivaroxaban was creatinine clearance of <50 mL/min/1.73 m^2^ (dose is reduced from 20 mg/day to 15 mg/day) and not recommended for use when renal function falls to <15 mL/min/1.73 m^2^ [21]. Dabigatran could be used at full doses till creatinine clearance of <30 mL/min/1.73 m^2^ [22]. Apixaban dose adjustments were multifaceted, which included considerations for serum creatinine, weight, and age [23]. For VTE management, full therapeutic doses of all DOACs were recommended until creatinine clearance of <30 mL/min/1.73 m^2^ (for rivaroxaban and dabigatran) and <25 mL/min/1.73 m^2^ (for apixaban) in which DOACs should be avoided since most VTE studies excluded these patients [[24], [25], [26]]. The differences in dose requirements for different indications accounted for 14% of dosing errors under “wrong dose for indication”, which was also encountered in other studies [17].

The study found disparity among prescribing patterns of apixaban in patients with advance renal disease (creatinine clearance < 15 mL/min/1.73 m^2^) or on dialysis. The incongruence in prescribing practice could be due to differences in dose recommendations by various authorities. Therapeutic use of apixaban in patients with end-stage renal disease or on dialysis was not approved by the Singapore licensing body, Health Sciences Authority (HSA). However, the use of apixaban in this population was approved by United States Food and Drug Administration (FDA) based on pharmacokinetic studies [27]. Pharmacist recommendations were based on local guidelines for apixaban dosing. However, some prescribers follow FDA recommendations and continue to prescribe apixaban in patients with advanced renal disease or on dialysis, while other prescribers would switch to an alternative anticoagulant.

Our study showed that the most encountered interventions among the prescribers were from cardiology and internal medicine, who were also the main prescribers for DOACs for patients with AF and VTE. However, our study did not investigate the prescribing discipline in the non-DRP group, which could affect the distribution of interventions among the disciplines. Another study conducted showed that clinicians from internal medicine had the highest rate of DOAC prescription followed by cardiology [28]. This also corroborated our findings, in which the same 2 disciplines contributed to the majority of DOACs prescribed among patients in the DRP group.

This study identified that impaired renal function and AF were factors that predisposed patients to DOAC-associated DRPs. However, Sell et al. found that number of concurrent medications and age were factors that predisposed a patient to DRPs. Unlike our study, this study focused on DRPs in general and not specific to DOAC therapy [29]. Other studies showed that elderly patients on DOACs for AF were at higher risk of bleeding compared with younger patients [30], had multiple comorbidities and higher mortality risk, and required drug therapy optimization to prevent poor outcomes [31]. Our study population was heterogenous, with patients on DOACs for multiple indications, and had wider age distribution, and hence, we did not find an association between age and comorbidities with DOAC-associated DRPs.

Poor renal function was significantly linked with DOAC-associated DRPs. When creatinine clearance decreased to >30 to 50 mL/min/1.73 m^2^, 15 to <30 mL/min/1.73 m^2^, and <15 mL/min/1.73 m^2^, there was an approximately 1.4, 2, and 2.4 times, respectively, increased likelihood of DRP detection compared with patients with creatinine clearance of >50 mL/min/1.73 m^2^. As DOACs rely on renal function for elimination, dose adjustment and increased monitoring are warranted to prevent accumulation of DOAC levels, which can lead to a bleeding event. Whittemore et al. showed that patients on DOACs with impaired renal function accompanied by other factors were associated with increased risk of bleed [32].

From our study, we found that patients on DOACs for AF were 1.8 times more likely to be associated with a DOAC-associated DRP compared with patients on DOACs for VTE. This could be due to inconsistency in prescribing patterns based on differences in local and overseas recommendations as well as complexity of dose adjustment for AF based on renal function for rivaroxaban; and age, weight, and serum creatinine for apixaban. Patients with AF were treated for a longer duration than those with VTE. Recommended duration for VTE was at least 3 to 6 months, while anticoagulation duration for AF was lifelong until benefits outweighed the risks. Hence, the longer duration of DOAC exposure for patients on AF, who are usually older and have multiple comorbidities, renal impairment, and higher pill burden, may increase their likelihood of DRPs and complications [31,33].

A systematic review showed that interventions to resolve DRPs can improve health outcomes and cost avoidance from healthcare utilization, even though results were not consistent across studies [34]. DOACs were classified as high alert medications and have healthcare cost implications resulting from thrombotic or bleeding events if prescribed improperly [35,36]. Addressing DOAC-associated DRPs and reducing prescribing errors may reduce risk of potential harm reaching patient, decrease preventable hospitalization events arising from DOAC-related complications, and reduce healthcare costs.

Our hospital practice to mandate all DOAC orders to be reviewed by pharmacists was similar to the hospital level safety surveillance implemented to oversee anticoagulant prescription under the Anticoagulation Stewardship program (ASP) described by Koolian et al. [37]. The ASP involved a dedicated team of physicians and clinical pharmacists who reviewed all anticoagulant prescriptions. Koolian et al. observed that 64% of all prescriptions generated interventions from ASP, which was higher compared with 9.8% in our study. The most common type of DRPs detected were inappropriate doses and drug interactions, which was comparable to our study. Intervention acceptance rates were higher at 84% compared with our study, which was 68.3%. Unlike our study, which looked only at DOACs, Koolian et al. included all types of anticoagulants in their surveillance, and this could account for the differences in DRP prevalence and acceptance rates.

Findings from this study, can provide development of focused improvement strategies to prevent prescription errors. Information technology (IT) enhancements to prompt renal dose adjustments, access to dose guides, and training are some strategies to improve prescribing habits. Studies showed that additional IT enhancements implemented to support the prescription of DOACs could lower the number of DRPs [38,39]. A qualitative study showed that improving the safety of DOAC use should be a multidisciplinary and multifaceted approach involving doctor, pharmacist, and patient and system level improvement strategies [40].

Our study had several limitations. The prevalence of DRPs could be underestimated since only electronically documented interventions were extracted. We were unable to collect verbal or manually documented interventions. Also, we could not rule out undetected DRPs nor patients with DRPs resolved from earlier prescriptions. Handling of missing data via mean substitution, which may lead to inconsistent bias and data replacement, may not reflect the true value. We did not perform a sensitivity analysis excluding patients with missing data. Being an observational study, patient adherence and concurrent medications were not examined. Although matching was performed to ensure that confounding factors were evenly distributed between the 2 groups, some residual confounders may remain. One of these factors was care setting because hospitalized patients tend to be more medically unstable compared with patients in outpatient settings and hence would encounter more DRPs. Our study but did not examine clinical outcomes such as bleeding and thrombosis or healthcare cost savings.

## 5 Conclusion

Common DOAC-associated DRPs were inappropriate drug regimen, avoidance of adverse events, and drug interactions. Factors that predispose patients to DOAC-associated DRPs were impaired renal function and AF. This study provides opportunities for implementation of focused system-level improvement strategies to reduce inappropriate DOAC prescription.
