# Supplementary material for: Drug-related problems associated with direct oral anticoagulants: an observational cross-sectional study of medical record review by pharmacists in a large teaching hospital
Source: Res Pract Thromb Haemost. 2024 Feb 15;8(2):102354. doi: 10.1016/j.rpth.2024.102354 (PMC10937962; doi:10.1016/j.rpth.2024.102354)
Supplement: Supplementary Tables [file mmc1.docx]

Appendix

Supplementary Table S1: Licensed Dosing Recommendations

| **Indication** | **Renal Function (CrCl ml/min)** | **Apixaban** | **Dabigatran** | **Rivaroxaban** |
| --- | --- | --- | --- | --- |
| **Atrial Fibrillation** | >50 | 5mg BD  2.5mg if patients for 2 or more or following: age >80 years, body weight <60 kg, SCr >133 umol/l | 150mg BD  110mg BD considered in high bleeding risk: eg. age >80 years, concurrent verapamil. Other considerations include moderate renal impairment, age 75-80 year, history of GI ulcers, or bleeding | 20mg OD |
|  | >30-50 |  |  | 15mg OD |
|  | 15-30 | 2.5mg BD | Avoid | Use with caution |
|  | <15 or dialysis | Avoid | Avoid | Avoid |
| **Venous thromboembolism** | >50 | 10mg BD for 7 days, then 5mg BD | 150mg BD after 5 days of parenteral anticoagulation  110mg BD 110mg BD considered in high bleeding risk: eg. age >80 years, concurrent verapamil. Other considerations include moderate renal impairment, age 75-80 year, history of GI ulcers, or bleeding | 15mg BD for 3 weeks then 20mg OM |
|  | >30-50 | 2.5mg BD for  Extended prophylaxis post 6 months treatment |  |  |
|  | 15-30 | Use with caution | Avoid | Use with caution |
|  | <15 or dialysis | Avoid use | Avoid | Avoid |
| **VTE prevention for hip or knee replacement surgery** | >50 | NA | 220mg OD for 10 days for knee replacement surgery, 28-35 days for hip surgery | 10mg OD |
|  | >30-50 | NA | 150mg OD |  |
|  | 15-30 | NA | Avoid | Use with caution |
|  | <15 or dialysis | NA | Avoid | Avoid |
| **Prevention of major cardiovascular events (CV death, MI and stroke) in** | >50 | NA | NA | 2.5mg BD with aspirin |
|  | >30-50 | NA | NA |  |
|  | 15-30 | NA | NA | Use with caution |
|  | <15 or dialysis | NA | NA | Avoid |
| **All indications** | Use in hepatic impairment | Contraindicated in:  Patients with liver disease with coagulopathy and clinically relevant bleeding risk, and severe liver impairment | Contraindicated in:   - Elevated liver enzymes >2x ULN - Hepatic impairment or liver disease | Contraindicated in:  Patients with hepatic disease associated with coagulopathy and clinically relevant bleeding risk including cirrhotic patients with Child Pugh B and C |

Supplementary Table S2: Modified PCNE DRP definitions

| DRP Categories | Definitions | Examples |
| --- | --- | --- |
| Inappropriate Drug Regimen | Patient has a medical problem being treated with a less than optimal regimen of the correct drug eg. disease state not responding, signs of toxicity, low or elevated serum drug level, inadequate or excessive duration of therapy and patient with unusual dosage requirement. | DOAC dose not adjusted for renal impairment.  Induction doses of rivaroxaban exceeding duration of 3 weeks. |
| Omission of Drug Therapy | Patient has a medical problem that requires drug therapy but is not receiving a drug for that indication or is not adequately controlled with drugs at optimal doses and requires additional drugs. | Patient with AF and high CHADSVASc not restarted DOAC post-operation when hemostasis was achieved |
| Improper Drug Selection | Patient has a drug indication but is taking an inappropriate drug eg. drug contraindication, drug unable to reach target site, inadequate response to the drug at optimal dose and require switch to alternative drug or evidence use of one drug over another. | DOAC used for off-label indication such as intra-cardiac thrombus, when warfarin is the effective alternative.  Rivaroxaban prescribed for AF, in a patient with end-stage-renal failure on dialysis, which is not recommended, and warfarin is a safer alternative |
| Therapeutic Duplication | Patient is taking more drugs than required, usually from the same therapeutic class, for the same indication. | Patient was switched from rivaroxaban to enoxaparin, but enoxaparin was ordered without discontinuing the rivaroxaban order |
| No Indication | Patient is taking a drug for no medically valid indication. | Patient with VTE with plans to be on anticoagulation for 3 months. The duration of rivaroxaban exceeded 3 months therapy, which will mean patient would anti- coagulated unnecessarily for a longer duration. |
| Avoidance of Adverse Event | Patient may have a medical problem that is the result of an adverse drug event which can be an extension of the drug’s pharmacologic effects of an allergic/idiopathic reaction. | Patient developed acute kidney injury while on rivaroxaban which can lead to drug accumulation and increase bleeding risk, if not held off or switched to heparin therapy |
| Therapeutic Substitution | The use of a chemically different drug that is therapeutically equivalent to, and often with similar toxicity profiles as the prescribed drugs. Includes IV-PO substitution and substitution due to drug unavailability and cost consideration. | Patient with cardioembolic stroke started on apixaban which was not in hospital formulary during that period, but rivaroxaban was. Suggested to switch to rivaroxaban. |
| Drug Interaction | Patient may have a medical problem that is the result of a drug-drug-food or drug-laboratory interaction. | Patient on rivaroxaban and started on rifampicin, a potent CYP450 isozyme inducer, which can reduce anticoagulation efficacy |
| Monitoring Parameters Recommendation | There is issue relating to investigations or parameters for monitoring adverse and therapeutic effects of drug therapy. | Patient with AF was switched from warfarin to rivaroxaban when INR was >2. Suggested to hold warfarin and recheck INR next day and when INR <2 start rivaroxaban. |
| Clarification of Drug Order | Order is ambiguous or incomplete and requires further clarification. |  |

Supplementary Figure S1: Patient Flow Chart


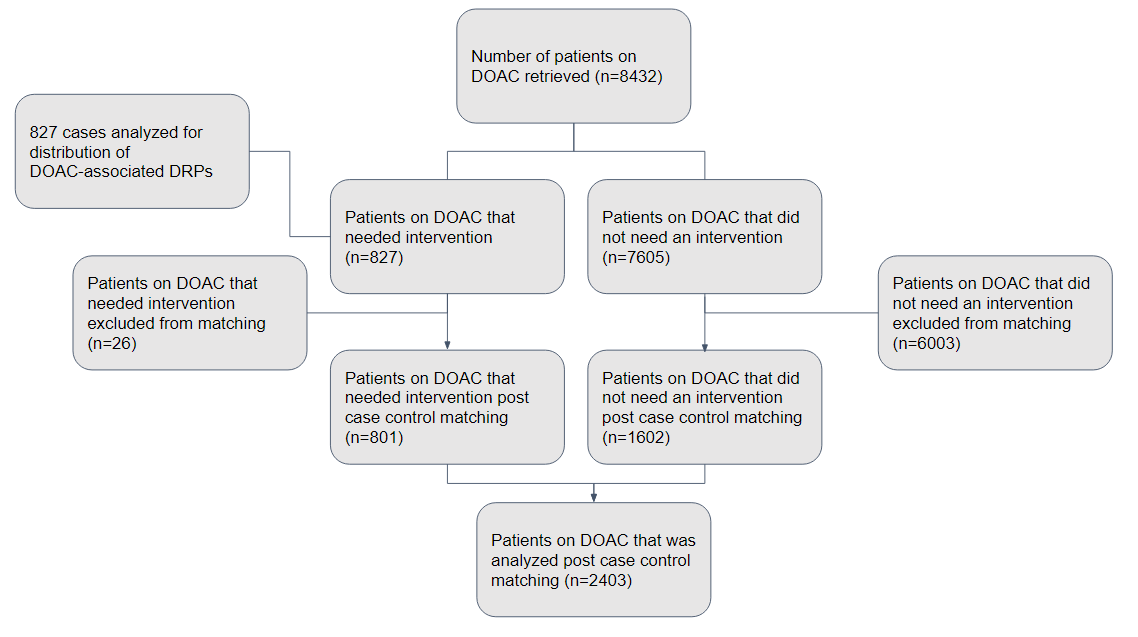


Supplementary Table S3: Distribution of Interventions based on care setting

|  | **Total n (%)**  **827** | | **Rivaroxaban**  **613 (74.1)** | | **Dabigatran**  **42 (5.1)** | | **Apixaban**  **172 (20.8)** | |
| --- | --- | --- | --- | --- | --- | --- | --- | --- |
| Inpatient | 760 | (91.9) | 560 | (73.7) | 35 | (4.6) | 165 | (21.7) |
| Outpatient | 67 | (8.1) | 53 | (79.1) | 7 | (10.4) | 7 | (10.4) |

Supplementary Table S4: Types of Pharmacist Recommendations

| **Recommendations** | **Total n (%)** | | **Rivaroxaban** | | **Dabigatran** | | **Apixaban** | |
| --- | --- | --- | --- | --- | --- | --- | --- | --- |
|  | **827** | | **613 (74)** | | **42 (5)** | | **172 (21)** | |
| Modify Dosage Regimen | 516 | (62.4) | 408 | (79.1) | 16 | (3.1) | 92 | (17.8) |
| Substitute Drug | 115 | (13.9) | 73 | (63.5) | 14 | (12.2) | 28 | (24.3) |
| Discontinue Drug | 82 | (9.9) | 59 | (72.0) | 4 | (4.9) | 19 | (23.2) |
| Initiate/Modify Monitoring or Investigations | 60 | (7.3) | 39 | (65.0) | 2 | (3.3) | 19 | (31.7) |
| Add Drug | 41 | (5.0) | 23 | (56.1) | 6 | (14.6) | 12 | (29.3) |
| Drug Information | 9 | (1.1) | 8 | (88.9) | 0 | (0.0) | 1 | (11.1) |
| Not documented | 3 | (0.4) | 2 | (66.7) | 0 | (0.0) | 1 | (33.3) |

Supplementary Table S5: Acceptance Rate of Recommendations

| Outcome | **Total n (%)** | | **Rivaroxaban** | | **Dabigatran** | | **Apixaban** | |
| --- | --- | --- | --- | --- | --- | --- | --- | --- |
| Accepted | 565 | (68.3) | 435 | (77) | 27 | (4.8) | 103 | (18.2) |
| Not Accepted | 128 | (15.5) | 79 | (61.7) | 10 | (7.8) | 39 | (30.5) |
| Accepted with modification | 93 | (11.2) | 70 | (75.3) | 3 | (3.2) | 20 | (21.5) |
| Not documented | 41 | (5) | 29 | (70.7) | 2 | (4.9) | 10 | (24.4) |

Supplementary Table S6: Logistic regression for unmatched population

| n=8,432 | **Unadjusted** | | **Adjusted** | |
| --- | --- | --- | --- | --- |
| **Factor** | **OR (95% CI)** | **p-value** | **AOR (95% CI)** | **p-value** |
| Age (years)  21-50  >50-65  >65-75  >75 | 1.00 (0.73 – 1.37) 0.96 (0.71 – 1.31) 1.31 (0.97 – 1.76) | 0.002 0.984 0.804 0.076 | 0.97 (0.70 – 1.32) 0.87 (0.64 – 1.19) 1.09 (0.80 – 1.50) | 0.110 0.825 0.390 0.587 |
| CrCl (ml/min/1.73m^2^)  >50  >30 to 50  15 to 30  <15 | 1.25 (1.06 – 1.47) 1.57 (1.26 – 1.97) 1.90 (1.09 – 3.32) | <0.001 0.008 <0.01 0.024 | 1.17 (0.98 – 1.40) 1.43 (1.12 – 1.82) 1.75 (0.99 – 3.08) | 0.011 0.081 0.004 0.053 |
| ≥ 2 Comorbidities  Yes | 0.84 (0.73 – 0.97) | 0.02 | 0.92 (0.79 – 1.08) | 0.302 |
